# Nonsurgical therapy for lumbar spinal stenosis caused by ligamentum flavum hypertrophy: A review

**DOI:** 10.1097/MD.0000000000038782

**Published:** 2024-07-05

**Authors:** Nan Fang, Zhigang Wang, Jiecheng Jiang, Aofei Yang, Tian Mao, Zitong Wang, Qian Chen

**Affiliations:** a College of Acupuncture & Orthopedics, Hubei University of Chinese Medicine, Wuhan, China; b Department of Orthopedics & Traumatology, Hubei Provincial Hospital of TCM, Wuhan, China; c Department of Orthopedics & Traumatology, Affiliated Hospital of Hubei University of Chinese Medicine, Wuhan, China.

**Keywords:** ligamentum flavum hypertrophy, lumbar spinal stenosis, nonsurgical treatment, research progress

## Abstract

Lumbar spinal stenosis (LSS) can cause a range of cauda equina symptoms, including lower back and leg pain, numbness, and intermittent claudication. This disease affects approximately 103 million people worldwide, particularly the elderly, and can seriously compromise their health and well-being. Ligamentum flavum hypertrophy (LFH) is one of the main contributing factors to this disease. Surgical treatment is currently recommended for LSS caused by LFH. For patients who do not meet the criteria for surgery, symptom relief can be achieved by using oral nonsteroidal anti-inflammatory drugs (NSAIDs) and epidural steroid injections. Exercise therapy and needle knife can also help to reduce the effects of mechanical stress. However, the effectiveness of these methods varies, and targeting the delay in LF hypertrophy is challenging. Therefore, further research and development of new drugs is necessary to address this issue. Several new drugs, including cyclopamine and *N*-acetyl-l-cysteine, are currently undergoing testing and may serve as new treatments for LSS caused by LFH.

## 1. Introduction

Lumbar spinal stenosis (LSS) often exhibits cauda equina symptoms such as lower back and leg pain, numbness, and intermittent claudication, and in severe cases, it can interfere with urinary and fecal elimination and even paraplegia.^[1]^ Epidemiological studies have shown that approximately 103 million people worldwide suffer from varying degrees of LSS, and the prevalence of the disease rises with age.^[2]^ Compression of spinal canal contents is a prerequisite for LSS. Previously, disc herniation was usually considered the primary cause of compression of the canal contents; however, according to imaging studies, while the spine is under load, the ligamentum flavum (LF) fills 50% to 80% of the spinal canal volume.^[3]^ The LF connects the upper and lower neighboring vertebrae and is essential for maintaining spinal stability. The LF adjacent to the central spinal canal, the intervertebral foramina, and the nerve root canals, so when ligamentum flavum hypertrophy (LFH) occurs, the LF compresses the aforementioned tissues, which have a dramatic effect on the athletic and sensory abilities of the lower extremities.^[4]^ Anatomical studies have indicated that the thickness of the normal lumbar LF is approximately 2 to 4 mm, and can be as high as 7 to 15 mm when LFH occurs, with varying degrees of calcification and ossification.^[5]^ Therefore, the inhibition of LFH, a pathological process, is crucial for the treatment of LSS.

Research has been conducted worldwide on the treatment of LSS caused by LFH. However, there is currently a lack of high-quality clinical protocols with clear evidence of LFH inhibition, except for surgical decompression.^[6]^ Moreover, surgical decompression is not suitable for all patients with LSS, as it can have a postoperative complication rate of up to 20%.^[7]^ For patients in the early clinical stage of LSS who require conservative treatment, available therapies primarily aim to provide analgesia, reduce inflammation, and alleviate mechanical stress. This text discusses nonsurgical treatments for LSS in the clinic and provides effective and practical methods as a reference for nonsurgical treatment of LSS. Owing to the absence of targeted treatments for LSS caused by LFH, we reviewed the drugs and therapies that are still in the experimental phase. These treatments mainly focus on inhibiting inflammation, fibrosis, and oxidation in LF to treat LSS, and have shown progress. This review provides ideas for the future development of new drugs.

## 2. Nonsurgical treatment of LSS

### 2.1. Anti-inflammatory and analgesic treatment of LSS

Modulation of cyclooxygenase-2 (COX-2) is an important pathway for improving neurological symptoms. The COX-2 inhibitor, celecoxib, reduces inflammation and relieves neuropathic pain caused by LSS.^[8]^ Celecoxib is an nonsteroidal anti-inflammatory drug (NSAID), and oral NSAIDs are currently an important therapy for early lower back pain and cauda equina symptoms in LS. Several NSAIDs including meloxicam and rifecoxib are similar to celecoxib. Acetaminophen is a safer alternative that is more commonly used in clinics for the elderly because of the need for a more comprehensive evaluation of the safety of NSAIDs.^[9,10]^ Hiroyuki et al^[11]^ discovered that oral acetaminophen administration resulted in a decrease of >0.8 points in the Zurich Claudication Questionnaire in 36.7% of patients with LSS, which was more effective than the exercise group. Furthermore, the study found that adding mirogabalin to NSAIDs further improved peripheral nerve pain in LSS without any new safety concerns.^[12]^ Apart from NSAIDs, Limaprost can enhance nerve function by increasing the blood supply to nerves.^[13,14]^ According to Japanese scholars Nikaido et al,^[15–17]^ Limaprost was found to be superior to etodolac (an NSAID) in improving symptoms, subjective satisfaction, and activity of daily living in patients with LSS. Combining Limaprost with Neurotropin may further improve lower back pain and lower extremity function.^[18]^

Since LSS often presents with lower back pain and radiating pain to the buttocks and legs, most therapies are analgesic and anti-inflammatory, such as oral NSAIDs. LSS with neurological claudication symptoms is recommended to avoid the above drugs as much as possible,^[19]^ and pregabalin may be a preferable option.^[20]^ Pregabalin can provide significant relief of peripheral nerve symptoms owing to its powerful analgesic ability; however, one should be aware of the many adverse events associated with pregabalin and the risk of discontinuing the drug for adverse events.^[21]^

For individuals who do not respond well to oral medications, a better option is epidural steroid injection (ESI), a method of improving radicular pain by injecting steroidal anti-inflammatory medications and local anesthetics into the epidural.^[22]^ ESI can rapidly improve pain and lower limb dysfunction due to LSS in the short term with a high degree of safety.^[23]^ In addition to ESI, steroid injections into the gluteal trigger point can significantly improve symptoms and quality of life in patients with LSS, with even better outcomes than ESI at 8 weeks and beyond.^[24]^ Furthermore, the injections are not only steroids, but also an autologous serum called gold-induced cytokines, which also provides good efficacy for its extremely anti-inflammatory properties.^[25]^

### 2.2. Anti-mechanical stresses treatment of LSS

Risk factors for LFH include mechanical stress, lumbar disc herniation, diabetes, obesity, age, and gender.^[26]^ Increased mechanical stress on the LF as a result of spinal segmental instability is now regarded as one of the major factors for LFH,^[27,28]^ and many available nonsurgical therapies mostly focus on decreasing tension in the LF and its surrounding soft tissues, such as needle knife, exercise, and manipulation.

Needle knife is a novel medical technique that pierces the body like a needle and cuts, peels, and loosens soft tissues inside the body like a knife. According to animal studies, ultrasound-guided needle knife performed on the LF of rabbit L7/S1 improves the function of the hindlimb and the disorganization of its LF fibers, as well as down-regulates the expression of transforming growth factor-β1 (TGF-β1) and angiopoietin-like protein 2.^[29]^ TGF-β1 has been reported to be one of the most prominent molecules causing collagen deposition,^[30]^ while angiopoietin-like protein 2 is closely associated with chronic inflammation in LF fibroblasts.^[31]^ Zhu et al^[32]^ treated an elderly patient with LSS due to LFH by needle knife under CT-guidance. The symptoms were relieved immediately after treatment, completely disappeared after 1 month, and did not recur during the one-year follow-up. The patient’s right LF at L2/3 and L3/4 became 1 mm thinner after treatment.

Regardless of the therapeutic regimen, there is consensus to work with functional exercises. Exercise therapy focusing on functional exercises for the lower back muscles can strengthen the core and reduce mechanical stress compensated by LF. Kim et al^[33]^ found that after 4 weeks of lumbar dorsiflexion training in 15 patients with LSS, the mean scores on the Oswestry Disability Index (ODI), Spinal Stenosis Scale, and Roland-Morris Disability Questionnaire decreased by >25%, indicating that the patients’ pain and lower limb function were obviously improved. Mu et al^[34]^ found that Japanese Orthopaedic Association scores and walking distance were improved in patients with LSS after core strength training such as plank, side plank, and bridge. Pauwels et al^[35]^ instructed 12 patients with LSS to perform bicycling, and 7 of them showed a mean decrease of 63.5% in the numerical rating scale after 3 months, suggesting marked relief of neurogenic pain.

In addition to these therapies, manipulative traction is also an effective means of treating LSS, which not only promotes the absorption of inflammatory cells but also relieves muscular spasm, thus reducing soft tissue tension and restoring the normal line of force to the spine.^[36]^ Using flexion-distraction techniques on localized spinal cord segments by manipulation can improve the patient’s VAS and ODI, but it requires a high level of skill on the part of the manipulator.^[37]^

### 2.3. Traditional Chinese medicine treatment of LSS

Acupuncture therapy involves inserting needles into the body to treat a disease. According to a randomized controlled trial conducted by Qin et al,^[38]^ patients in the acupuncture group experienced faster restoration of lower limb function and pain relief than the non-insertive sham acupuncture group, in patients with LSS. However, a study suggested that acupuncture does not significantly improve pain and lower limb function in patients with LSS compared with routine care.^[39]^ Therefore, larger trials with better design are required to validate the efficacy of acupuncture. Electroacupuncture therapy is a technique derived from traditional acupuncture that involves the dual stimulation of needles and electricity through the application of trace low-frequency pulsed currents close to the bioelectricity of the body. A study in animals found that electroacupuncture can activate neuronal networks across multiple systems and produce anti-inflammatory effects.^[40]^ Additionally, when combined with manipulation and exercise therapy, it can provide maximum pain relief and improve lower limb function.^[41]^

In addition to acupuncture, traditional Chinese medicine has gradually become the mainstream method of nonsurgical treatment for LSS in China. Buyang Huanwu Decoction was originally used as a classic formula for the treatment of poststroke sequela, and it can also have a positive therapeutic effect on LSS caused by LFH. Network pharmacological studies have found that the Buyang Huanwu Decoction can affect up to 50 therapeutic targets in LF, with a complex and diverse mechanism.^[42]^ Li et al^[43]^ found that Buyang Huanwu Decoction supplemented with extracts from rabbit skin inflamed by vaccinia virus improved the VAS and Japanese Orthopaedic Association scores of patients with LSS and reduced the incidence of complications such as rash and vomiting. Furthermore, Shujin Jianyao Pills and Tongdu Huoxue Decoction have potent anti-inflammatory and analgesic properties. These properties can decrease the expression of various inflammatory mediators, such as TGF-β1, TNF-α, IL-6, and IL-1β in the serum, and alleviate pain.^[44–47]^

Most of the above nonsurgical therapies have outstanding efficacy in patients with only neurogenic symptoms without degenerative spondylolisthesis or scoliosis and with a disease duration of <1 year.^[48,49]^ This approach is generally based on pharmacological treatments, which will continue to be the primary method for conservative treatment of LSS in the future. However, only a limited number of drugs are available, with symptom reduction as the primary focus, drug target limited to only a few targets such as COX-2, and even fewer studies on LSS caused by LFH. Except for a case study that clearly indicated that needle knife can thin hypertrophic LF, none of the other therapies have shown clear evidence of improving LFH, so potential drug targets and corresponding treatment strategies need to be developed.

## 3. Potential therapeutic strategies of LSS

### 3.1. Potential anti-inflammatory strategies of LSS

Chronic inflammation, mediated by inflammatory mediators such as IL-6, IL-1β, and TNF-α, is considered a significant factor contributing to LFH,^[50]^ the search for therapeutic targets to regulate inflammation in LF is imperative, among which periostin may become a new potential anti-inflammatory target. Periostin is an extracellular matrix (ECM) protein, and Akito et al^[51]^ discovered that overexpression of periostin increased the expression of IL-6 in human and rabbit LF, whereas inhibition of periostin decreased the expression of IL-6. Periostin may also play a role in transforming LF cells into myofibroblasts and in overexpressing collagen, which are key factors leading to histological changes in LFH.^[52–54]^

Leptin is also closely associated with IL-6, and leptin can upregulate the expression of IL-6 through the activation of the NF-κB signaling pathway leading to LF inflammation.^[55,56]^ It is worth noting that leptin was originally a hormone secreted by adipose tissue, and leptin levels in human serum rise with increasing body fat,^[57]^ suggesting that obesity is one of the factors in LFH, which shows that the inhibition of leptin expression and weight loss are important for alleviating LF inflammation. In the field of medicinal chemistry, modulators that inhibit leptin expression have been exploited but have not yet been applied to the treatment of LSS.^[58,59]^

### 3.2. Potential antifibrotic strategies of LSS

**Figure 1.**
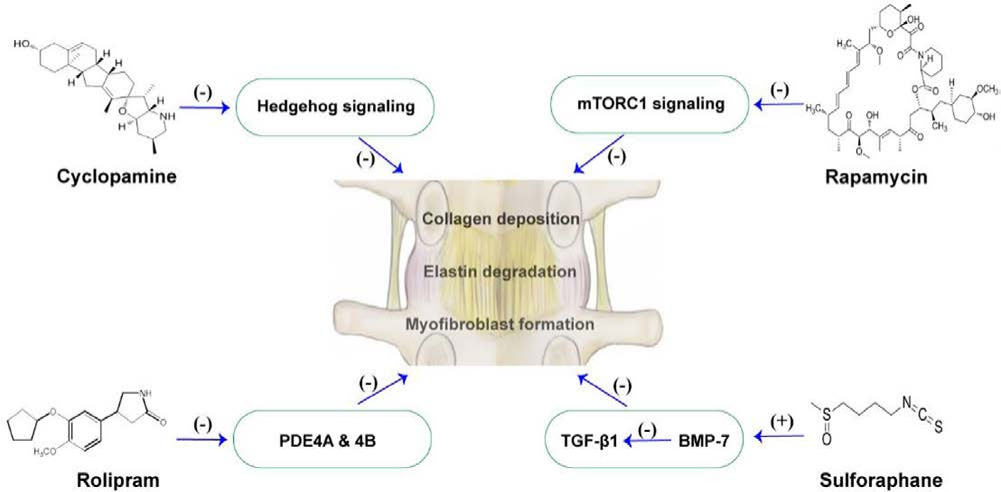
The main pathways and their representative drugs in the antifibrotic treatment of ligamentum flavum hypertrophy. BMP-7 = bone morphogenetic protein-7, mTORC1 = mammalian target of rapamycin complex 1, PDEs = phosphodiesterase, TGF-β1 = transforming growth factor-β1.

Histological studies suggest that LFH is characterized by LF fibrosis resulting from the rebuilding of cells and ECM. This involves a series of processes, such as collagen deposition, elastin degradation, and myofibroblast formation.^[26,60,61]^ Inhibiting these processes through antifibrotic therapy is an effective strategy for improving LSS caused by LFH (Fig. 1).

Cyclopamine, a steroidal alkaloid, plays an important role as a specific antagonist of the Hedgehog (Hh) signaling pathway in cancer, fibrosis, and other areas.^[62]^ The Hh signaling pathway is also an important pathway contributing to LF fibrosis,^[63]^ and therapeutic administration of cyclopamine can play a positive role in the down-regulation of collagen expression, inhibition of fibroblast proliferation and differentiation, and even in subsequent ossification of LF by inhibiting this pathway.^[64]^ In addition, cyclopamine has antioxidant activity in disc degeneration and significantly enhances the survival of nucleus pulposus cells,^[65]^ but there are no studies on its antioxidant effects in LF. Evidently, the dual antifibrotic and antioxidant properties of cyclopamine may have a positive impact on the inhibition of LFH.

The mammalian target of rapamycin complex 1 (mTORC1) and Hh signaling pathways are important for cancer therapy. Research has shown that mTORC1 pathway is closely linked to LF fibrosis.^[66]^ Mechanical stress promotes the production of insulin-like growth factor-1 (IGF-1) by LF cells, which in turn promotes the synthesis of type 1/3 collagen (COL-1/3) through the IGF-1R/Akt/mTORC1 pathway. Currently, COL-1/3 is considered the major collagen involved in ECM reconstruction in LFH.^[61]^ Rapamycin, an mTOR inhibitor, reduce COL-1/3 synthesis by inhibiting this pathway.^[67]^ Rapamycin, also known as sirolimus, is an immunosuppressant with strong anti-inflammatory and antifibrotic properties.^[68]^ Previous research has shown that rapamycin inhibits TGF-β1 signaling, hindering the growth and proliferation of fibroblasts.^[69]^ This finding is valuable for future LFH research.

Phosphodiesterases (PDEs) typically mediate fibrosis and inflammatory responses in various systemic diseases.^[70–72]^ PDEs 4A and 4B were highly expressed in hypertrophied LF. The use of rolipram, an inhibitor of PDE4, not only significantly inhibited PDE4 activity but also suppressed the expression of Col I/III, fibronectin, and TGF-β1, indicating that rolipram may exert an antifibrotic effect in LFH by inhibiting PDE4 activity.^[73]^ Rolipram attenuates fibroblast activity and suppresses proliferation and differentiation.^[74]^ Additionally, it has neuroprotective properties and may exert antiallodynic effects.^[75,76]^ These findings are crucial for the treatment of lower limb neurological symptoms of LSS. In summary, the antifibrotic and antiallodynic effects of rolipram make it a promising treatment option for LSS.

TGF-β1 is an essential molecule leading to LF fibrosis, and TGF-β1 exerts its fibrosis-stimulating effects via the Smad2/3, PI3K/Akt, β-catenin, and ERK1/2 signaling pathways.^[2,30,73]^ It is feasible to inhibit LF fibrosis by modulating the concentration and potency of TGF-β1 in the serum and LF tissues. Previous studies have shown that bone morphogenetic protein-7 (BMP-7) interferes with TGF-β1 signaling and expression of various matrix proteins, thereby inhibiting ECM remodeling.^[77]^ It has been suggested that BMP-7 could be a potential target for reversing TGF-β1.^[78,79]^ Sulforaphane, a dietary agent, can prevent diabetes-induced renal fibrosis by upregulating BMP-7 expression and subsequently inhibiting TGF-β1 signaling.^[80]^ Similarly, artemisinin and its derivatives may also exert antifibrotic effects by increasing the expression of BMP-7.^[81]^

### 3.3. Potential antioxidant strategies of LSS

Inflammation and fibrosis have a tremendous impact on LFH, but at the same time, the effects of oxidative stress cannot be disregarded. Epidemiological studies have shown that the incidence of LSS increases with increasing age. As the body ages, its ability to counteract oxidative stress decreases, coupled with increased mechanical stress in the LF, resulting in the generation of excess reactive oxygen species (ROS) that cannot be scavenged. This imbalance ultimately leads to a decrease in cellular activity, structural changes, and an increase in the rate of apoptosis.^[82]^ Furthermore, oxidative stress activates the Akt/MAPK signaling pathway, which exacerbates inflammation and fibrosis in LF.^[2]^ Therefore, antioxidants are necessary to inhibit LFH.

The Keap1/Nrf2/ARE antioxidant pathway was found to be an important signaling pathway for the inhibition of LFH,^[83]^ and N-acetyl-L-cysteine (NAC)-derived carbonized polymer dots activate this pathway to inhibit the production of ROS for the treatment of periodontitis.^[84]^ NAC is a precursor of glutathione, which is converted to glutathione in vivo^[85]^ and subsequently inhibits the expression of many oxidative and fibrotic markers and inflammatory mediators in hypertrophic LF cells, attenuating the adverse effects of oxidative stress on LF.^[86,87]^ Moreover, inflammation of the cauda equina and nerve roots is often accompanied by LSS,^[88]^ and NAC attenuates the long-term inflammatory response of the nerves and the toxic effects induced by free radicals, suggesting that NAC may be protective against the inflammatory nerve roots and cauda equina in LSS.^[89,90]^

Similar to NAC, harpagophytum procumbens (HP) also plays an antioxidant role via the Nrf2/NQO-1 signaling pathway. HP is a widespread medicinal plant in South Africa that has long been used as an ideal anti-inflammatory and analgesic for a variety of conditions including Alzheimer’s disease and rheumatoid arthritis.^[91]^ However, HP also has powerful antioxidant properties. HP extract has been shown to increase the expression of antioxidant pathway molecules such as nrf2 and HO-1, decrease the expression of oxidative stress molecules such as iNOS and COX-2, and eliminate ROS in the spinal cord of LSS rats, thereby promoting neuronal synapse repair.^[92,93]^ The structure of ethyl acetate in HP not only prevents oxidative stress in vitro but also modulates dopaminergic neurotransmission. Thus, it is a viable drug for the treatment of motor abnormalities.^[94]^ It is currently being investigated for the treatment of lower limb motor dysfunction in LSS. Commercial tinctures have been prepared from the roots of plants and have potent anti-inflammatory, analgesic, and antioxidant properties.^[95]^ Pharmacokinetic studies in horses have shown that HP extract has no detectable side effects.^[96]^

### 3.4. Other potential treatment strategies of LSS

LSS often results in pathological manifestations of spinal cord and nerve root injury.^[97]^ Human umbilical cord mesenchymal stem cells derived exosomes (HUCMSC-EVs) have good reparative properties for neural tissues, and HUCMSC-EVs were previously found to accelerate the regeneration of rat sciatic nerve after transection.^[98]^ HUCMSC-EVs have been applied in various regenerative fields because of their powerful tissue repair and regeneration capabilities.^[99]^ In addition to their excellent regenerative properties, HUCMSC-EVs can also be used as drug delivery vehicles. It has been found that HUCMSC-EVs can carry miR-146a-5p to promote spinal cord functional recovery by targeting neurotoxic astrocytes, which is critical for the repair of chronic spinal cord injury in LSS.^[100]^ Cheng et al^[101]^ for the first time, used HUCMSC-EVs as a vehicle to deliver 2 miRNAs, miR-146a-5p and miR-221-3p, to inhibit LFH by suppressing the TGF-β/SMAD4 signaling pathway. In addition to miR-146a and miR-221, miRNAs that exert antifibrotic and anti-inflammatory effects in LFH, such as miR-29a and miR-10396b, may have beneficial effects.^[102,103]^ HUCMSC-EVs can also treat experimental nonalcoholic steatohepatitis by activating the Nrf2/NQO-1 antioxidant pathway,^[104]^ which is one of the important antioxidant pathways in LFH.^[83]^ Thus, studying the delivery system of HUCMSC-EVs would be valuable for treating LSS.

Recent studies have found a positive correlation between amyloid deposition of transthyretin (TTR) and the thickness of the LF and epidural fat.^[105,106]^ Maurer et al^[107]^ detected amyloid deposits in the LF of >1/3 of patients with LSS, and in more than half of these patients the precursor of the deposited amyloid was TTR. Drugs targeting TTR to inhibit amyloid deposition for treating fibrotic diseases have emerged, such as Tafamidis, which can bind to TTR and prevent tetramer dissociation and amyloidogenesis.^[108]^ In the clinic, Tafamidis has been used to treat TTR amyloid cardiomyopathy,^[109]^ but not LSS caused by LFH.

## 4. Perspectives

In summary, there are several ways to treat LSS caused by LFH. ESI, exercise therapy, and needle knife have demonstrated clear efficacy and are widely used in clinical practice. However, there is still a lack of targeted drugs that can inhibit LFH. Several drugs targeting LF for treating LSS are currently being developed and tested, among which cyclopamine, rapamycin, and NAC are likely to be more valuable in terms of research and application and may become new drugs for the treatment of LSS caused by LFH in the future.

## Author contributions

**Conceptualization:** Nan Fang.

**Data curation:** Jiecheng Jiang, Zitong Wang.

**Investigation:** Zitong Wang.

**Supervision:** Qian Chen.

**Writing – original draft:** Nan Fang.

**Writing – review & editing:** Zhigang Wang, Aofei Yang, Tian Mao.
